# Engineering an Optogenetic pH‐Modulator in Bacteria

**DOI:** 10.1002/advs.202524319

**Published:** 2026-05-07

**Authors:** Jenevieve Kuang, Olivia J. Armendarez, Wei‐Ting Chang, Matthew M. Hausladen, Shanna Bonanno, Daniel J. Wilson, Neel S. Joshi, Leila F. Deravi

**Affiliations:** ^1^ Department of Chemistry and Chemical Biology Northeastern University Boston Massachusetts USA; ^2^ Kostas Research Institute Northeastern University Burlington Massachusetts USA; ^3^ Department of Bioengineering Northeastern University Boston Massachusetts USA; ^4^ Department of Chemical Engineering Northeastern University Boston Massachusetts USA

**Keywords:** deployable living systems, engineered living material, light‐responsive bacteria, optogenetics, pH modulation

## Abstract

Cells in many naturally occurring organisms routinely cooperate to control their extracellular pH in a dynamic and reversible manner, but this capability has been underexplored in synthetic biology. Here, we sought to engineer a microbial system that switches between two states —high and low extracellular pH— with minimal human intervention. We accomplished this by combining: (1) a genetic circuit that produces recombinant urease under the control of a light‐inducible promoter; (2) a degradation tag on urease to accelerate the high‐to‐low pH transition; and (3) optimization of several environmental factors, including media composition, replenishment rate, and light exposure patterns. The system raises the pH when urease is produced and hydrolyzes urea in the media to produce ammonia; it lowers the pH as a byproduct of the cell's native metabolism when urease production ceases. We demonstrate that the optimized system cycles continuously for up to 14 days with minimal performance loss. Overall, our system demonstrates synthetic pH control in an engineered living system and highlights challenges and potential solutions for using such systems outside of the context of typical laboratory manipulation.

## Graphical abstract

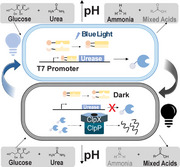

## 1 Introduction

The field of synthetic biology has greatly expanded the range of tasks that microorganisms can perform through rational engineering, including executing complex logic operations [1, 2], dynamically regulating gene expression [3, 4], and modulating environmental parameters such as pH [5, 6]. Progress in the development of Engineered Living Materials (ELMs) intended for practical applications includes examples like bioplastics that can be triggered to auto‐degrade after a period of use through the action of embedded microorganisms [7], or textiles whose pigmentation can be programmed by living cells during the fabrication process [8]. These useful advances demonstrate engineered cells as active participants in either the manufacture (as in the textile example), or the end‐of‐life processes (as in the bioplastic example), but not during the main period of use of either material. In contrast, cells in natural living systems participate continuously in sense‐and‐respond, maintenance, actuation, and other functions.

Some progress has been made toward more deployable versions of engineered cellular systems, including strategies to maintain metabolic activity [9, 10], preserve the fidelity of genetic engineering [11, 12], and prevent cellular escape into the environment through encapsulation in hydrogels [13, 14] or miniaturized bioreactors [15]. But, major challenges remain in developing systems that operate outside of conventional laboratory environments (37°C, enriched media, and shaking cultures). For instance, the performance of genetic circuits is often demonstrated in cultures that are being agitated constantly to facilitate oxygen transfer, ensure homogeneous nutrient distribution, and prevent cell sedimentation [16, 17]. These conditions support a high growth rate and high metabolic activity, which in turn increases the rate of protein synthesis and degradation, making them ideal for generating unidirectional input‐to‐output function (e.g., input of small molecules yields an output of fluorescent proteins). More complex behaviors, like reversible switching between states, are more difficult to control, especially in non‐traditional culture conditions, without interventions that require altering the media composition, like the use of chemical inducers.

**SCHEME 1:**
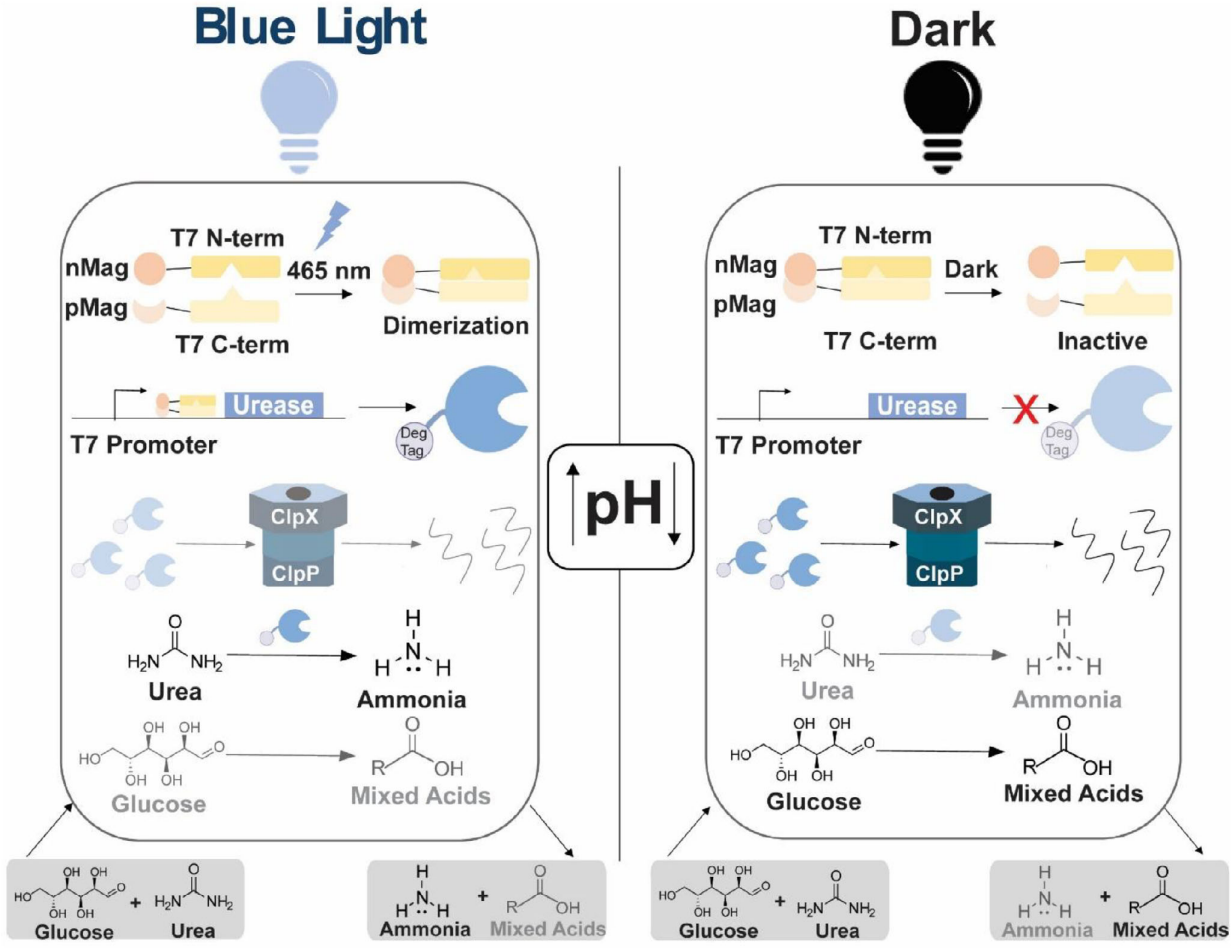
Mechanism of blue light‐induced pH modulation in *E. coli*. Upon blue light exposure, T7 RNA polymerase dimerizes, initiating the transcription of urease. Urease catalyzes the hydrolysis of urea into ammonia, resulting in an increase in the pH of the surrounding medium prior to protein degradation. Urea‐driven alkalinization dominates in situ culture pH when compared to glucose metabolism and protein production. In the absence of blue light, T7 RNA polymerase reverts to its inactive form, halting urease production. Protein degradation reduces intracellular urease concentrations and slows urea hydrolysis. Glucose metabolism dominates culture pH by producing acidic byproducts. A portion of the graphical elements in the schematic were created in Biorender; Joshi, N. (2026) https://BioRender.com/0cnmy7u.

Our goal was to demonstrate that engineered living systems, powered by a steady replenishment of nutrients, could modify their material surroundings repeatedly in a programmed manner. We viewed this as a stepping stone toward more autonomous engineered living systems capable of stably responding to cues provided by an external user without requiring the more invasive manipulations typically used for microbial culture and maintenance in the laboratory. For our demonstration, we developed a pH modulation platform using engineered *E. coli* that enables dynamic environmental control without the need for exogenous chemical inducers. Prior work from our laboratory demonstrated that microbially‐driven reversible pH changes can mediate material size and color transformations [18]. This work advances this concept in several ways. First, we incorporated a recombinant urease gene under the control of a photo‐responsive split T7 RNA polymerase (Scheme 1). Urease catalyzes the hydrolysis of urea provided in the growth media, producing ammonia, which basifies the extracellular environment. This allows the system to access a wider range of pH values (between 4.5 and 9.0) and trigger basification without chemical induction. Second, we tuned the degradation rate of the urease enzyme to accelerate the transition from high to low pH, which also depends on the ability to produce acidic byproducts from the native *E. coli* metabolism. When urease activity decreases, endogenous *E. coli* metabolism generates acidic byproducts that drive re‐acidification of the environment. Thus, a full pH reversal (high → low) occurs autonomously within a single culture without media replacement. While individual high‐to‐low transitions do not require media exchange, repeated cycling over extended durations may necessitate media replenishment to restore substrate availability and maintain system performance. Finally, we show that precise tuning of environmental conditions—urea concentration and light exposure—together with nutrient replenishment enables reliable switching between high and low pH states over multiple cycles with minimal performance loss.

## 2 Results

### 2.1 Tuning the Production and Degradation of the Urease Enzyme Complex

To explore conditions that enable the semi‐autonomous function of cells, we first surveyed optogenetic switches and their function in *E. coli* strain BL21(DE3). We started with a two‐component kinase‐based system originating in *Synechocystis*, UirS/R [19], which has been demonstrated to control the transcription of target genes in response to blue light irradiation [20]. When we introduced urease genes under the control of the UirS/R system, light irradiation did raise the pH of the cultures; however, leaky expression in the absence of light made it difficult to decrease the pH in our system (Figure S1). Based on these data, we suspected that either the lack of a repressor in the two‐component system or crosstalk between native kinases and UirR was responsible for the leaky expression [21]. To mitigate this crosstalk, we explored an alternative optogenetic system that controlled RNA polymerase function directly using a split T7 RNA polymerase (Opto‐T7). To avoid transcription of urease from the chromosomal T7 RNA polymerase in *E. coli* strain BL21(DE3), experiments were conducted in *E. coli* strain DH10B. In this system, each half of the polymerase is fused to either side of a split “Magnet” domain—“nMag” and “pMag” [22]. Upon blue light irradiation, nMag and pMag undergo dimerization, mediated by a flavin chromophore. This reconstitutes the full T7 RNA polymerase from the fused fragments and activates transcription.

**FIGURE 1:**
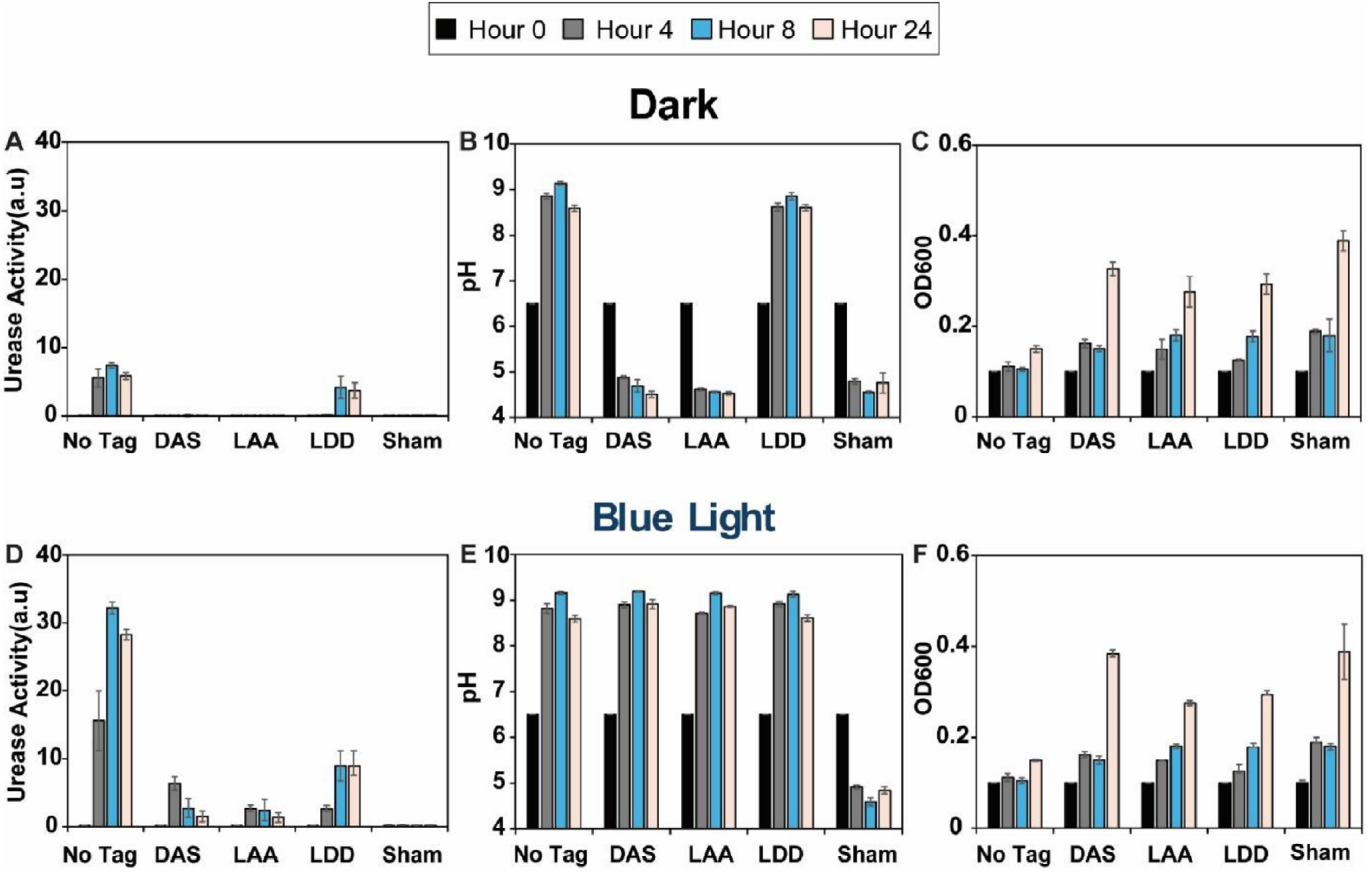
Effects of degradation tags on urease activity, in situ culture pH, and cell growth. (A) Urease activity from dark control. (B) In‐situ culture pH from dark control. (C) Cell density from dark control. (D) Urease activity from 4 h of blue‐light exposure. (E) In situ culture pH from four hours of blue‐light exposure. (F) Cell density from 4 h of blue‐light exposure. Error bars indicate standard deviation (*n* = 3). Statistical tests are plotted in Figure S5.

Initially, we observed that the re‐engineered system in which urease expression was driven by Opto‐T7 was also leaky, leading to a significant pH increase after the light was turned off (Figure S2A). A measurement of urease activity independently from in situ culture pH indicated that the irradiated cells produced enzyme; however, activity levels continued to rise for more than 24 h post irradiation (Figure S2B). This suggested that residual urease inside the cells was sufficient to maintain a high pH of the culture condition due to continued ammonia production after irradiation was over. To address this leaky expression, we evaluated three degradation tags fused to one of the urease subunits (UreB) with varying degrees of recognition by the ClpX machinery: LDD (no affinity), DAS (moderate affinity), and LAA (high affinity) (Table S1) [23]. To test the effects of the degradation tags on urease activity, we irradiated cells under blue light for four hours and monitored enzyme production over 24 h. We used defined media in this system to ensure reproducibility and reduce batch‐to‐batch variation in buffering capacity. We found that cells engineered to produce these urease variants exhibited different enzyme lifetimes and the ability to transition from a high to low pH state (Figures 1A‐F, Figure S5). LDD exhibited only a slight change in urease activity compared to the “no‐tag” variant under dark conditions, whereas DAS and LAA variants showed no basal urease activity, presumably because these tags increased the UreB degradation rate to be higher than any leaky production (Figure 1A). The in situ pH of the cultures reflected their measured urease activity in the dark prior to any light exposure, with the “no‐tag” and LDD cultures rising from pH 6.5 to 9.1 and those for DAS and LAA dropping to pH 4.5 after four hours (Figure 1B). The pH drop for DAS and LAA occurred by design, since we provided excess glucose in the culture medium and expected its conversion into mixed acids as a byproduct of glycolysis [24]. All of the cultures exhibited relatively higher urease activity after four hours of continuous blue light irradiation (correlating to a pH increase to 8.90), except for the one bearing the “Sham” plasmid that did not encode any urease genes (Figure 1D). After the four‐hour post‐irradiation period, the urease activity decreased in the DAS and LAA cultures but increased in the “no‐tag” and LDD cultures, validating our hypothesis about the effect of the degradation tags. Despite the observed changes in urease activity, the in‐situ culture pH remained high for all cultures (Figure 1E). We attribute this to the bacteria's weak ability to metabolize ammonia, although it should be noted that *E. coli* natively possesses pathways for ammonia assimilation [25]. All cultures showed lower OD_600_ values over the course of the experiment compared to the Sham control, suggesting that the metabolic burden of producing the enzymes and the high pH conditions of the culture negatively impacted cell growth rate (Figure 1C,F; Figure S5).

### 2.2 Using Light Dosage to Tune Bidirectional pH Transitions

Using the strains producing UreB fused to the DAS degradation sequence, which we will refer to as “Urease‐DAS,” we next explored experimental conditions to achieve a reversible living pH modulator. Using a custom‐built adjustable irradiation system, we evaluated the effects of light intensity and duration on the urease expression and pH of our system (Figure S3A). We began by exploring the wavelength selectivity of the construct. Here, we irradiated Urease‐DAS cultures with either green (550 nm), red (650 nm), or blue (450 nm) light for 1 h with a fixed irradiance of 6 mW/cm^2^ and a cumulative light dosage of 21.6 J/cm^2^ (Table S2). As expected, only cultures exposed to blue light exhibited an increase in pH from a starting value of 5.6 to 9.0 after 3 h, with the pH remaining high up to 24 h. Cultures exposed to green or red light showed a pH decrease from 6.5 to 4.5 over the course of 24 h (Figure S3B). The in situ culture pH values were in line with the corresponding urease activity. The cultures exposed to blue light showed a sharp increase in urease activity over the first three hours and a subsequent decrease after 24 h (Figure S3C).

**FIGURE 2:**
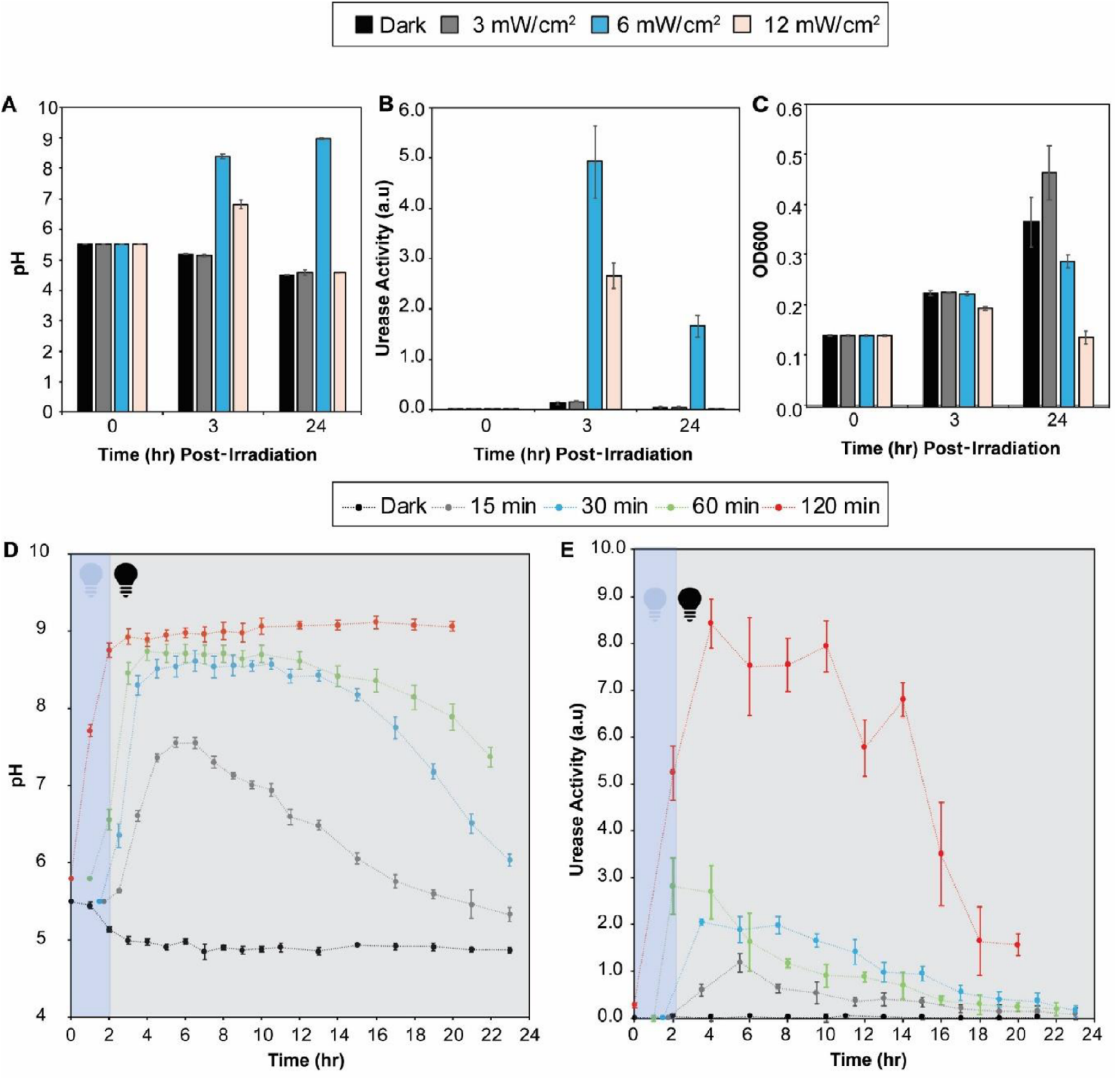
Effect of light dosage on in situ culture pH and urease activity over time. The changes in (A) pH, (B) urease activity, and (C) bacterial growth were measured against varied blue‐light irradiance with fixed light durations of 1 h, with time points being measured at 3‐h and 24‐h post‐irradiation. Likewise, the (D) pH kinetics and (E) urease activity of Urease‐DAS cultures were monitored with higher time resolution and a wider range of light exposure times, suggesting variable acidification and basification rates. All bars and scatter dots indicate mean ± standard deviation (*n* = 3).

To assess the effect of light intensity on modulating enzymatic activity, we fixed the duration of light exposure to one hour, while testing various blue‐light irradiance values—0 mW/cm^2^, 3 mW/cm^2^, 6 mW/cm^2^, or 12 mW/cm^2^—resulting in light dosages of 5.4 J/cm^2^, 10.8 J/cm^2^, and 21.6 J/cm^2^ (Table S2). All cultures had a starting pH of 5.6 and an initial urease activity value of 0.01 a.u. For the dark condition and the low intensity condition, the pH was acidified 24 h post‐irradiation to pH 4.5 and pH 4.6, respectively (Figure 2A). Additionally, minimal urease activity was observed in those same conditions (Figure 2B). For the cultures exposed to moderate and high light intensity, pH increased three hours post‐irradiation to 8.4 and 6.8, respectively (Figure 2A). 24 h after irradiation, these cultures had pH values of 8.9 and 4.5, respectively (Figure 2A). The urease activity of the cultures again qualitatively reflected the in situ culture pH, although high light intensity conditions returned to basal activity levels after 24 h, while the moderate conditions did not (Figure 2B). Cell density measurements during the experiment showed that cultures exposed to higher light intensities grew more poorly (Figure 2C), possibly suggesting that photocytotoxicity may place an upper bound on the range of intensities that could be used in future experiments while maintaining high cell viability.

To further elucidate the minimal induction of urease activity that would enable cultures to both rise to pH 9.0 and then re‐acidify to pH 4.5 as fast as possible, we investigated the lower limit of light duration, moving forward with a fixed irradiance of 6 mW/cm^2^. Table S3 contains a light dosage matrix that identifies the irradiance, light exposure duration, and calculated light dosage for each condition, along with their associated acidification and basification rates. In Figure 2D, the samples exposed to blue light for the longest durations—1 h and 2 h, receiving cumulative doses of 21.6 J/cm^2^ and 43.2 J/cm^2^—raised the surrounding media to above pH 8.5 within 2 h of irradiation. The highest (2‐hour) dosage had the quickest basification rate of ~1.5 pH units per hour (Table S3), yet pH remained high over the course of 22 h, resulting in no re‐acidification. Similarly, the highest dosage led to the highest urease activity at the same time point (Figure 2E). The 1‐h dosage had a slightly lower basification rate, yet pH returned to neutral within 22 h following light exposure (Figure 2D). The 30‐min dosage exhibited a similar basification rate as the higher dosages, and reached a comparably high pH, while returning to pH 5.7 after 24 h (Figure 2D). The 15‐min dosage reached a maximum pH of only 7.5 before returning to pH 5.2 at 22 h. Overall, acidification rates were slower than basification rates, even though urease activity often returned to basal levels long before the cultures began to re‐acidify. This suggests that the native ammonia metabolism and glycolytic acidification mechanisms were the rate‐limiting process in the kinetics of the high‐to‐low pH transition of our system, not the enzyme activity within the cells.

**FIGURE 3:**
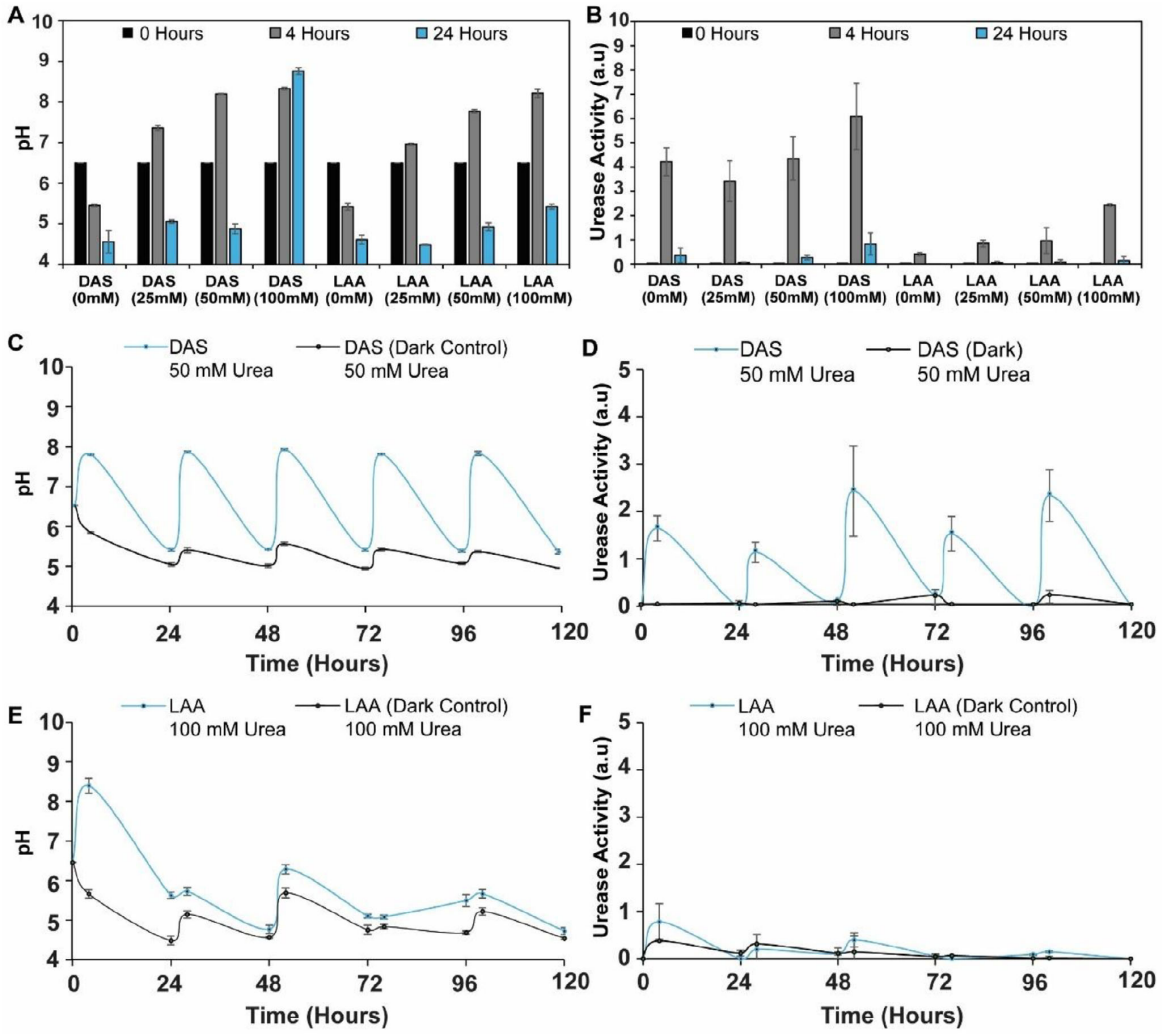
Investigating the effects of urea concentration on system longevity over multiple cycles of nutrient replenishment. Time course of (A) pH and (B) urease activity at four (solid) and 24 h (dashed) with previously optimized light exposure (30 min of blue 6 mW/cm^2^) for DAS‐tagged and LAA‐tagged constructs at various total urea concentrations. Cycling experiment showing (C) pH and (D) urease activity with data points taken every four and 24 h after nutrient replenishment for Urease‐DAS cultures. (E, F) Analogous data presented for LAA‐tagged cultures. Bars indicate mean ± standard deviation (*n* = 3). Statistical tests are plotted in Figure S6.

We next attempted to expand the experiment to cyclic pH modulation. By combining the light exposure time (30 min) that optimally raised and lowered pH with a screen of degradation tags (Figure S4), we observed that the culture pH of all urease variants remained high (Figure S4F), even though a comparable experiment (Figure 2D) showed re‐acidification. We rationalized that since we were replenishing media every time a data point was taken in the latter case (Figure 2D,E), the cells were provided sufficient substrate to continuously lower the pH of the media. Without that replenishment, our system appeared unstable, leading to some experiments where the cells were unable to overcome the harsh conditions. To further optimize the system for repeated cycling and decrease this element of unpredictability, we investigated two strategies: (1) reducing the total urea substrate available to the cells to convert to base, and (2) supplying a continuous flow of nutrients to support the reacidification process. Initially, we screened a range of lower total urea concentrations to identify conditions that maintained an in‐situ culture pH peak of above 8, followed by reacidification to pH <5. We found that the Urease‐DAS constructs were able to meet these requirements at a concentration of 50 mM urea, whereas the Urease‐LAA construct could not basify as efficiently (Figure 3A,B). To investigate the stability of our genetic engineering mechanisms and longevity of our system, we supplied the cells with optimized conditions (Urease‐DAS; 6 mW/cm^2^ blue light for 30 min) and observed their ability to cyclically basify and reacidify their environment under static culture conditions with nutrient replenishment once every 24 h. We found that both the in situ culture pH (Figure 3C) and urease activity (Figure 3D) showed reliable behavior over five full cycles. In contrast, analogous experiments run with the non‐optimal Urease‐LAA tag showed breakdown of the system after a single full cycle (Figure 3D,E; Figure S6). For these experiments, we selected urea concentrations that showed a pH rise above 8 after four hours (50 mm for DAS, 100 mm for LAA).

### 2.3 Continuous Nutrient Flow to Enhance System Switching Stability

**FIGURE 4:**
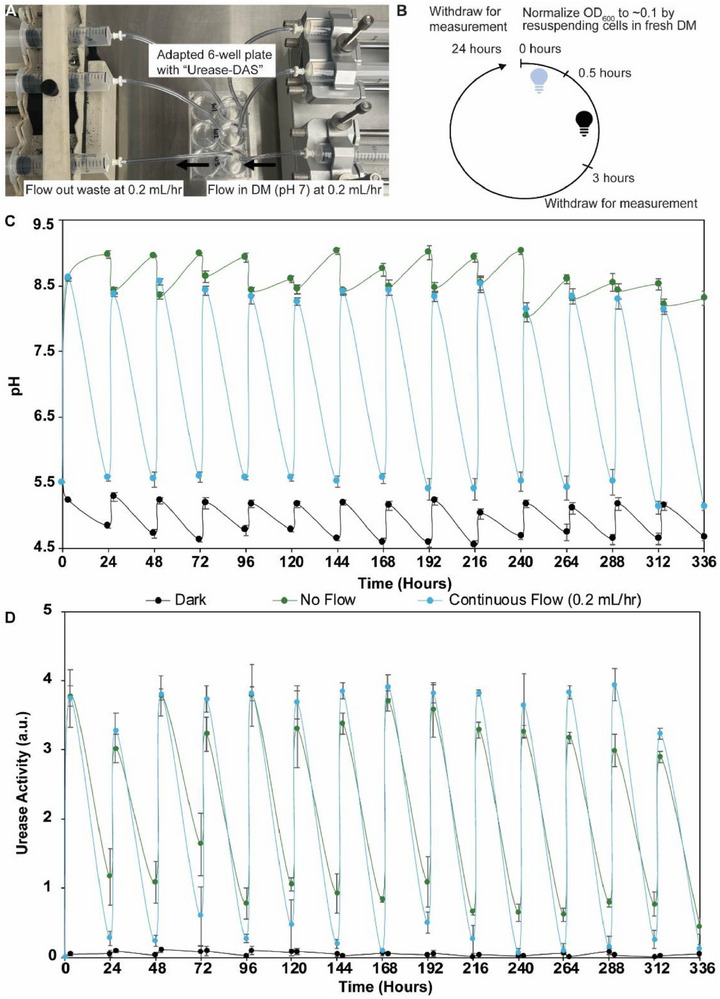
Effects of continuous media flow on culture pH cycling. (A) Schematic showing a well‐plate adaptation and its connectivity for continuous flow. Syringe pumps flowed in fresh media and removed culture from the well at the rate of 0.2 mL/hr, maintaining a constant volume in the wells. The full volume of the culture vessel was exchanged 28 times over 336 h. (B) An overview of the light exposure schedule and aliquot removal used for analysis. (C) In situ culture pH values and (D) corresponding urease activity values for three conditions through 14 days of basification and reacidification. Data indicate mean ± standard deviation (*n* = 3).

In parallel with lowering the urea concentration, we also investigated supplying a continuous flow of fresh nutrients to the system to enhance the speed and reliability of the high‐to‐low pH transition. We rationalized that after reaching their peak pH values, cells may have exhausted all or most of their glucose supply, making it difficult for them to reacidify through glycolysis. Continuous flow could help alleviate this problem by replenishing the glucose supply throughout each cycle while maintaining a constant volume. We set up a continuous flow system wherein a syringe pump supplied defined medium to the cultures in well‐plates at a constant flow rate of 0.2 mL/hr. Another syringe pump removed culture volume at a matched flow rate (Figure 4A). For the experimental setup, cultures were initialized at pH 5.5, and OD_600_ = 0.1, and we used other conditions optimized from previous iterations of the project (Urease‐DAS constructs, 30‐min light exposure, 6 mW/cm^2^ light dosage, 100 mM urea). The first cycle started with a defined medium at pH 5.5 and transitioned from light to dark conditions after 30 min. During continuous flow of pH 7.0 medium through the wells, aliquots removed from the wells at 3 h and 24 h after the start of each cycle were used to characterize pH, urease activity, and cell density (Figure 4B–D; Figure S7). After the first cycle, we observed that the cell density had reached OD_600_ values of 0.5 – 0.8, significantly higher than the ∼0.3 values we observed for static cultures (Figure S7). In separate experiments, we observed that high cell densities led to an inability to produce urease and basify the system (Figure S8). We believe the inability of higher cell densities to produce urease and basify the system might be due to their rapid consumption of nutrients in the media. Therefore, we opted to “reboot” the system after each 24‐hour full cycle to normalize the cellular density, allowing us to modulate pH reliably over many cycles in this configuration (Figure S9).

We found that cultures under continuous flow could cycle reliably from pH 5.5 to pH 8.0 (or higher) and back again over 24 h for 14 days (Figure 4C). The highest measured in situ culture pH values decreased from 8.5 to 8.0 after day 10 of the experiment, perhaps pointing toward a limit to how long the living system could endure the constant oscillation between pH extremes while maintaining the fidelity of the genetically encoded programs. We ran two control experiments in which comparable cultures were grown without continuous flow—either in continuous darkness or with the same light exposure schedule. As expected, the cultures grown in darkness always acidified their environment, which was consistent with the low basal urease activity measured for the samples (Figure 4C,D). The culture that received the same periodic light exposure, but did not have media flow during the cycles, showed culture pH values that were consistently above 9.0, at the mid‐cycle timepoint (Figure 4C). Since these cells were pelleted and resuspended in fresh media just like the experimental condition, the pH appears to cycle only between 8.5 to 9.0. This supports our hypothesis that, without a fresh supply of glucose, the cells were unable to recover from the harsh basic pH conditions.

## 3 Conclusion

We engineered a living system to alter the pH of its surroundings in a dynamic, yet predictable manner through a combination of genetic engineering and environmental controls. We demonstrated that the system could cycle between extreme pH values reliably for 14 days with minimal human intervention. This required significant optimization of several factors, including the type of light‐induced transcriptional control of the urease enzyme, optimization of the degradation rate of the enzyme, and identification of a minimum light dosage and urea concentration. All these factors were necessary to prevent cell death or malfunction even under conditions that negatively impacted long‐term cell growth and viability. Our system did require a “reboot” every 24 h (Figure 4) to prevent cellular overgrowth in the simple continuous flow adaptation of our well plates. We attempted to increase the flow rate to prevent this overgrowth, but cell sedimentation in the wells prevented our flow setup from diluting them effectively (Figure S9). We think it is possible to address this issue with further device engineering to prevent sedimentation.

The type of pH cycling we demonstrate may be useful in several practical contexts. Controlling pH under non‐native conditions has been proposed as a mechanism to influence the porosity and degradation of materials used for controlled drug release and targeted therapy [26, 27], end products in bioproduction [28], and environmental remediation [29]. This platform has potential applications in bioremediation. An example of this is, while the optimal growth pH of most microorganisms is around 7, the optimal pH range for remediation by chemical precipitation of heavy metals such as copper, zinc, and lead is around 9 [30]. Our system creates an opportunity to use engineered organisms to actively adjust native environmental pH to conditions favorable for chemical precipitations while resetting the pH to one that is favorable for growth. While microbial pH control systems have been reported previously, to our knowledge, none have demonstrated reversible, deployable pH modulation suitable for operation outside of traditional culture environments.

Further work will be necessary to advance such systems toward practical application. For example, other work from our lab developing color‐ and shape‐changing hydrogels and miniaturized bioreactor devices could help convert this system into a continuously operating sensor or device that responds to its biochemical surroundings [18]. More complex genetic circuits could also be implemented to produce additional pH‐modulating enzymes, such as esterases, to actively lower the pH of the system using parallel optogenetic systems. Beyond the challenges addressed in this work, further studies are required to resolve issues related to nutrient flow, waste management, and long‐term genetic stability. For example, stress associated with elevated ammonia levels and extreme pH could pose challenges for certain applications. However, overexpression of ammonia assimilation pathways could help reduce ammonia levels in the system more rapidly [31]. Overall, this work shows that the engineering of living systems that are intended for complex switching behavior and environmental modulation requires a holistic analysis and optimization of genetic and design parameters.

## 4 Methods

### 4.1 Strains and Plasmids

Cell strains used in this study are listed in Table S4. Plasmids used in this study are listed in Table S5. pOpto‐T7 Urease variants were constructed using the urease amino acid sequences (Table S6) from a previously reported plasmid, pBR322‐Ure. pOpto‐T7 was constructed using genes encoding the urease enzyme complex (*ureABCD…*) isolated from *Sporosarcina pasteurii*. Genes were inserted into the pAB50 plasmid backbone (Addgene) using PCR amplification followed by NEB Gibson Assembly cloning kit. pOpto‐T7 Urease variants were then transformed into One Shot Mach T1 chemically competent *E. coli* cells following the provider's protocol. Constructs were isolated using the QIAprep Spin Miniprep Kit and sequenced using nanopore sequencing (Twist Biosciences). pOpto‐T7 Urease degradation tag variants were synthesized using Twist Biosciences, followed by the cloning steps stated above.

### 4.2 Media Composition

Luria‐Bertani (LB) media was prepared using 10 g of tryptone, 5 g of yeast extract, and 10 g of NaCl dissolved in 1 L of distilled water. Defined media (DM) preparation was modified for increased buffering capacity. It contained 0.2 mm CaCl_2_ 2H_2_O,0.5% (w/v) of CAS amino acids, 1 mm MgCl_2_ H_2_O, 3.2 µm thiamine, 41 µm K_2_HPO_4_, 8.0 mM NaCl, 2.0 mm KCl, 2.0 mM NH_4_Cl, 0.4 µM FeCl_2_, and 0.3 mMm Na_2_SO_4_ in distilled water.

### 4.3 Cell Culture

Cultures were inoculated from single colonies of the transformed *E. coli* pOpto‐T7 Urease variants and were grown overnight in the dark with LB media containing carbenicillin (100 µg/mL) and chloramphenicol (33 µg/mL) in a shaking incubator at 37°C and 225 rpm. The overnight culture was centrifuged at 4,000 RCF for 10 min at 4°C. Unless otherwise noted, the cellular pellet was resuspended in DM supplemented with 100 µM nickel (II) chloride, 100 mM urea, 2% (w/v) glucose, carbenicillin (100 µg/mL), and chloramphenicol (33 µg/mL).

### 4.4 Characterizing Degradation‐Tag Urease Variants in Single Cycle

Strains were normalized to 0.1 OD_600_ in DM supplemented with 100 µm nickel (II) chloride, 100 mM urea, 2% (w/v) glucose, carbenicillin (100 µg/mL), and chloramphenicol (33 µg/mL). Cells were aliquoted into 12‐well plates. Cells were induced using 80 W LED flood lights with a fixed irradiance of 6 mW/cm^2^ at 37°C. 200 µL samples of culture were collected at various time points to measure pH, OD_600_, and urease activity.

### 4.5 Light Dosage Calculations

The irradiance (mW/cm^2^) was experimentally measured using a ThorLabs optical power and energy meter console (spot size: 1 cm^2^) with a fixed distance between the sample and the light source. To mitigate any irradiance loss from the air between the liquid samples and the lid of the 6‐well plate, the lid was placed ∼3 cm above the detector when measuring irradiance values. The light dosage (J/cm^2^) was calculated using the following equation:

(1) $$\mathrm{Dosage}\;\left(\frac{J}{cm^{2}}\right)=\frac{Irradiance\;\left(\frac{mJ}{\sec*cm^{2}}\right)\times \;Time\;\left(sec\right)}{1000}$$

### 4.6 Measuring Urease Activity with Phenol Red Colorimetric Assay

Phenol red (10 mg/L) in 1 X Phosphate‐Buffered Saline (PBS), pH 6.8, was sterile‐filtered using a 0.2 µm membrane. Experimental samples were washed three times with the phenol red solution using a 0.2 µm filtered 96‐well plate. The cells were diluted with the phenol red solution to a final OD_600_ of ∼0.10. A 180 µL aliquot of the diluted cells was added to a 20 µL aliquot of 200 mg/mL in phenol red solution. The change in OD_560_ was monitored every minute for 1 h using a Spectra Max M5 spectrophotometer as a modified protocol to a previous method [32]. The slope changes from OD_560_ measurements, denoted by y, were used in a linear standard curve to extrapolate urease activity (a.u.), denoted by x, in (2).

(2) y=0.0008x−0.0026

### 4.7 Characterizing Wavelength Selectivity of Urease‐DAS

The Urease‐DAS samples were irradiated using an LED light source of 450 nm blue, 550 nm green, and 650 nm red light for 60 min with a fixed irradiance of 6 mW/cm^2^. A 400 µL aliquot of the samples was used for pH, OD_600_, and urease activity testing pre‐irradiation, 3‐h post‐irradiation, and 24 h post‐irradiation. The volume loss was replaced with a 400 µL aliquot of supplemented DM pH 7.0.

### 4.8 Variable Blue Light Irradiance on Urease‐DAS

The Urease‐DAS samples were placed under an LED light source with 465 nm blue light using variable irradiance ranging from 0, 3, 6, and 12 mW/cm^2^ for a fixed duration of 1 h. For dark controls of 0 mW/cm^2^, the well plates were covered from light exposure using aluminum foil. Following the appropriate light dosage, samples were kept in the dark in a static incubator at 37°C. A 400 µL aliquot of samples from the varied blue light intensities was taken for subsequent testing of pH, OD_600_, and urease activity testing pre‐irradiation, 3 h post‐irradiation, and 24 h post‐irradiation. Aliquots taken out were replenished with supplemented DM pH 7.0.

### 4.9 Variable Blue Light Exposure Times on Urease‐DAS

The Urease‐DAS samples were placed under a 465 nm blue light LED light source with a fixed irradiance of 6 mW/cm^2^ and varied durations of light exposure ranging from 0 min, 15 min, 30 min, 1 h, and 2 h. For dark controls (0 mW/cm^2^), the well plates were shielded from light. Following the appropriate light dosage, samples were kept in the dark in a static incubator at 37°C. A 400 µL aliquot of samples from the varied blue light intensities was taken for subsequent testing of pH, OD_600_, and urease activity testing pre‐irradiation, and at various time points post‐irradiation. Aliquots taken out were replenished with supplemented DM pH 7.0.

### 4.10 Characterizing Degradation‐Tag Urease Variants in Multiple Cycles Without Continuous Flow

Strains were normalized to 0.1 OD_600_ in DM supplemented with 100 µm nickel (II) chloride, 100 mM urea, 2% (w/v) glucose, carbenicillin (100 µg/mL), and chloramphenicol (33 µg/mL). Cells were aliquoted into 12‐well plates, then induced using 80 W LED flood lights with a fixed irradiance of 6 mW/cm^2^ at 37°C for 30 min before the lights were shut off. 200 µL of the sample culture was taken out at 4 h to measure pH, OD_600_, and urease activity. After 24 h, cell cultures were aspirated out, and 200 µL samples of cell culture were collected and used to measure pH, OD_600_, and urease activity. New cultures were made by replenishing supplemented DM media pH 6.8 to the remaining cells in each of the wells. Fresh cultures were immediately induced using blue light for 30 min at 37°C and remained in incubation for 4 h before 200 µL of the sample culture was collected. This process occurred daily for 5 days. For dark controls (0 mW/cm^2^), the well plates were shielded from light.

### 4.11 Cycling of Minimum Blue‐Light Activation Dosage on Urease‐DAS

The following pulse sequence was used every 24 h, which we consider 1 complete cycle. The Urease‐DAS samples were placed under a 465 nm blue light LED light source with a fixed irradiance of 6 mW/cm^2^ for 30 min, with subsequent aliquots taken for measuring at 3 h post‐ and 24 h post‐irradiation. The condition without continuous flow only received replenishment of supplemented DM pH 7.0 when aliquots were withdrawn for testing. Alternatively, another condition had constant withdrawal of the sample and infusion of supplemented DM pH 7.0 at flow rates of 0.2 mL/hr using an adapted well plate lid. At the start of a cycle following initial measurements, cells were spun down using a centrifuge at 4,000 RCF for 10 min. To mitigate the consequence of overgrowth on performance, the cellular pellet was resuspended in supplemented DM pH 5.5 to an OD_600_ of 0.10. For dark controls (0 mW/cm^2^), the well plates were shielded from light. Following the appropriate light dosage, samples were kept in the dark in a static incubator at 37°C.

### 4.12 Statistical Analysis

Pre‐processing of data was limited to normalization and conversion of reaction rates into arbitrary units for urease activity. Statistical analyses of pH, OD, and urease activity were carried out using GraphPad Prism 8. Data are expressed as mean ± SD. The sample size (n) for each experiment is reported in the corresponding figure legends. Unless otherwise stated, Student's t‐test was used when comparing two groups as indicated in the legend. For all experiments, replicates consisted of a starter culture that originated from a single microbial colony partitioned into multiple wells.

## Conflicts of Interest

The authors declare no conflict of interest.

## Supporting information




**Supporting File**: advs75492‐sup‐0001‐SuppMat.docx.

## Data Availability

The data that support the findings of this study are available in the supplementary material of this article.
