# Supplementary material for: Engineering an Optogenetic pH‐Modulator in Bacteria
Source: Adv Sci (Weinh). 2026 May 7;13(42):e24319. doi: 10.1002/advs.202524319 (PMC13335442; doi:10.1002/advs.202524319)
Supplement: Supplementary file 1 — Supporting File: advs75492‐sup‐0001‐SuppMat.docx. [file ADVS-13-e24319-s001.docx]

**Engineering an Optogenetic pH-Modulator in Bacteria**

**Supporting Information**

*Jenevieve Kuang^*^, ^1^ Olivia J. Armendarez^*^,^1^ Wei-Ting Chang, ^1^ Matthew M. Hausladen,^2^ Daniel J. Wilson, ^2,3^ Neel S. Joshi, ^1*^ and Leila F. Deravi^1*^*

*These authors contributed equally to this work.


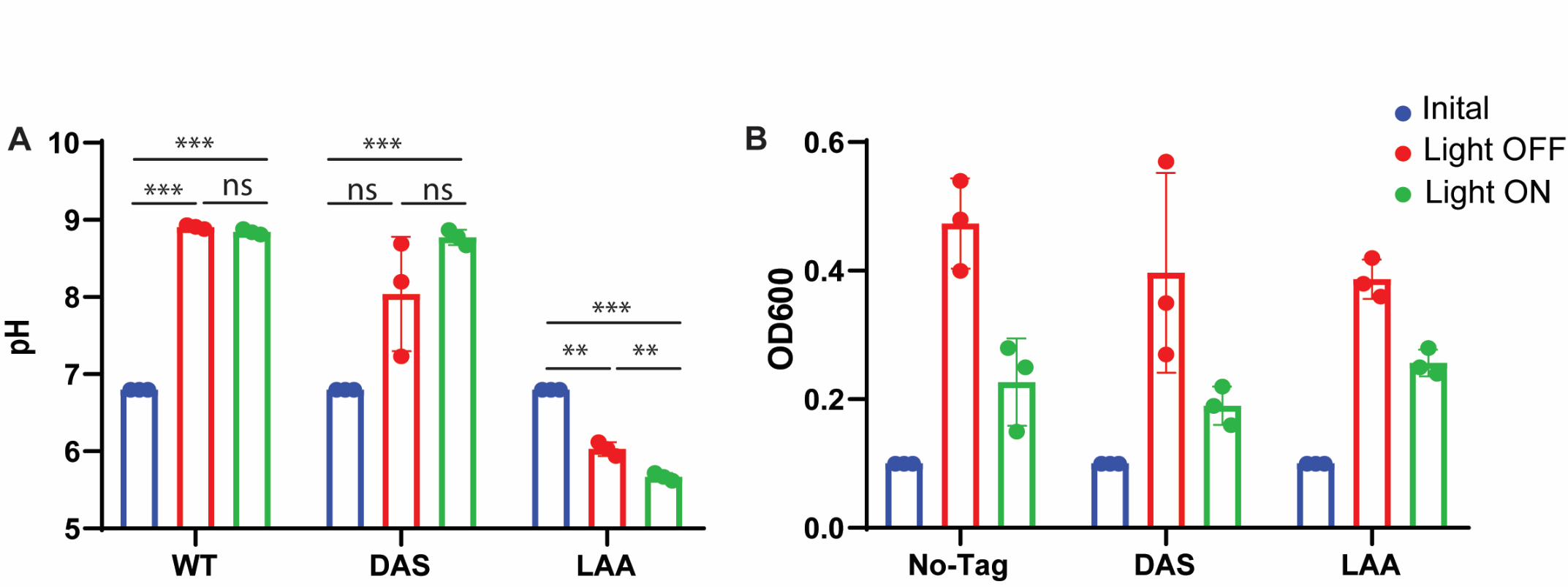


**Figure S1.** Leaky urease expression in UirR/UirS. **A**) pH of Initial, “Light Induction-OFF” and “Light Induction-ON” state of UirR/UirS Urease **B**) OD_600_ of Initial, ‘Light Induction-OFF” and “Light Induction-ON” state of UirR/UirS Urease. A paired two-tailed t-test was used to determine statistical significance between two groups groups (n = 3, ns, ∗p > 0.05, ∗∗p < 0.01, ∗∗∗p < 0.001). Bars indicate mean ± standard deviation (n=3) replicates derived from the same starter culture.


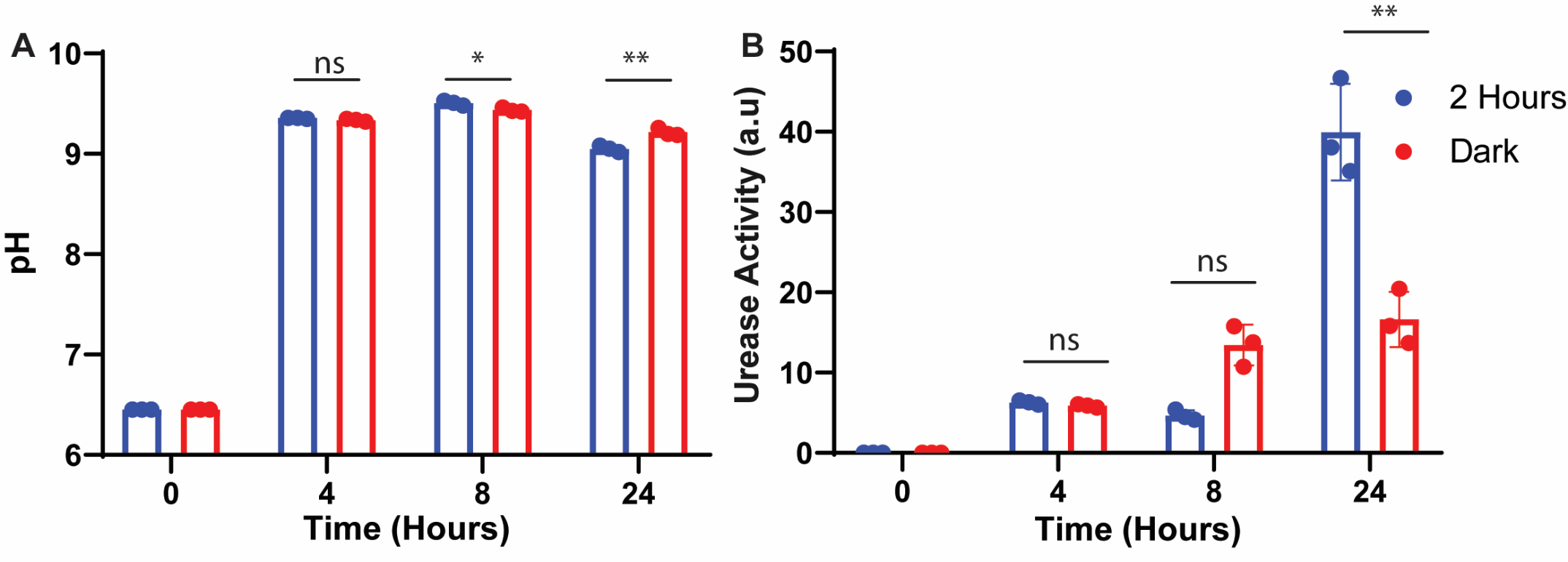


**Figure S2.** Leaky expression of urease controlled by the Opto-T7 induction system. **A**) In-situ culture pH for cultures that have either been induced for 2 hours or kept in the dark using *E.coli* strain transformed with pOpto-T7 encoding urease with no degradation tag. **B**) Urease activity measured independently from in situ culture pH for the same cultures as in (**A**). A paired two-tailed t-test was used to determine statistical significance between two groups (n = 3, ns, ∗p > 0.05, ∗∗p < 0.01). Bars indicate mean ± standard deviation (n=3) replicates derived from the same starter culture.

**Table S1.** Panel of degradation tags used to control urease concentrations.

| **Variant Name** | **Affinity for Degradation Machinery** | **Amino Acid Sequence** |
| --- | --- | --- |
| No-Tag Variant | None/Very Low | N/A |
| DAS Tag | Moderate | AANDENYADAS |
| LAA Tag | High Affinity | AANDENYALAA |
| LDD Tag | Very Low | AANDENYSENYALDD |


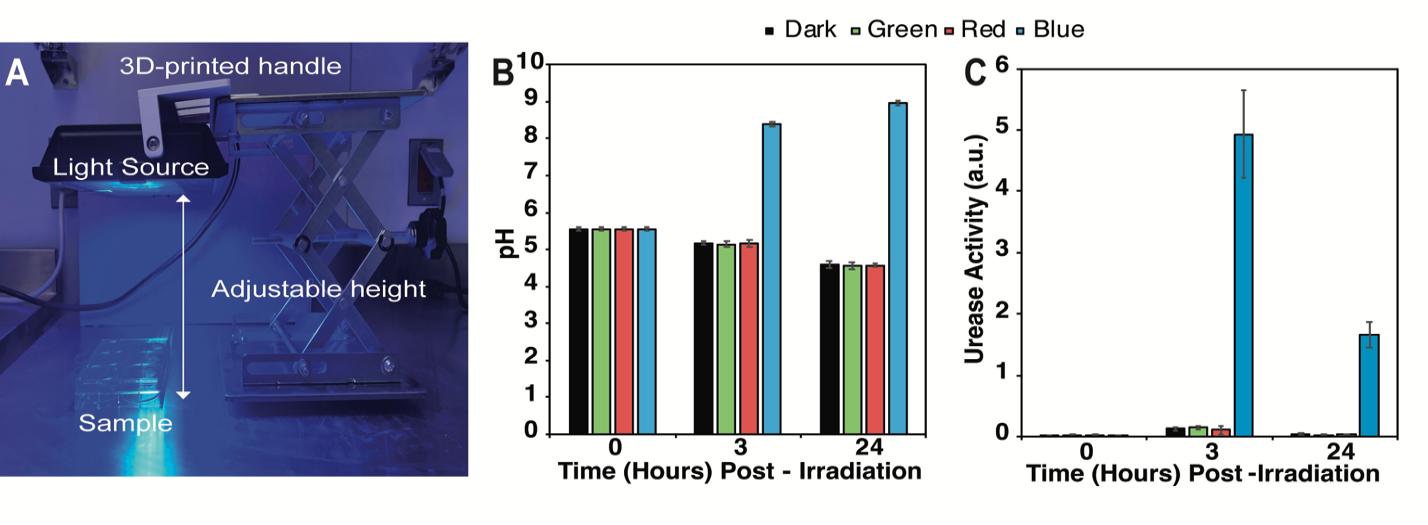


**Figure S3:** **A**) Image of illumination setup with adjustable irradiance based on distance between the sample and the light source. **B**) Blue-light selectivity of Opto-T7 system as measured by in situ culture pH for *E. coli* expressing Urease-DAS irradiated for 1 hour with green-, red-, and blue-light induction at 6 mW/cm^2^ for 24 hours. **C)** Urease activity (ng/mL) measured independently from in situ culture pH for the same cultures as shown in (**A**). Bars indicate mean ± standard deviation (n=3) replicates derived from the same starter culture.

**Table S2.** Illumination parameter table highlighting the wavelength of the light source, distance from light source to sample, irradiance, time, and calculated light dosage.

| **Figure** | **Light Source**  **Wavelength (nm)** | **Distance from light source (cm)** | **Irradiance (mW/cm^2^)** | **Time**  **(minutes)** | **Light Dosage (J/cm^2^)** |
| --- | --- | --- | --- | --- | --- |
| 1 | Blue (465) | 10 | 9 | 240 | 129.6 |
| S3,2 | Blue (465) | 19 | 6 | 60 | 21.6 |
| S3 | Green (550) | 19 | 6 | 60 | 21.6 |
| S3 | Red (650) | 19 | 6 | 60 | 21.6 |
| 2 | Blue (465) | 30* | 3 | 60 | 10.8 |
| 2 | Blue (465) | 9.5 | 12 | 60 | 43.2 |
| 2 | Blue (465) | 19 | 6 | 120 | 43.2 |
| 2-4 | Blue (465) | 19 | 6 | 30 | 10.8 |
| 2 | Blue (465) | 19 | 6 | 15 | 5.4 |

*Used irradiance rather than distance to decrease intensity

**Table S3.** Acidification and basification rates of Urease-DAS based on varied light dosages.

| **Irradiance (mW/cm^2^)** | **Time**  **(minutes)** | **Light Dosage (J/cm^2^)** | **Acidification Rate (pH/hr)** | **Basification Rate (pH/hr)** |
| --- | --- | --- | --- | --- |
| 6 | 120 | 43.2 | 0.00 | +1.5 |
| 6 | 60 | 21.6 | -0.1 | +1.3 |
| 6 | 30 | 10.8 | -0.2 | +1.1 |
| 6 | 15 | 5.4 | -0.1 | +0.6 |


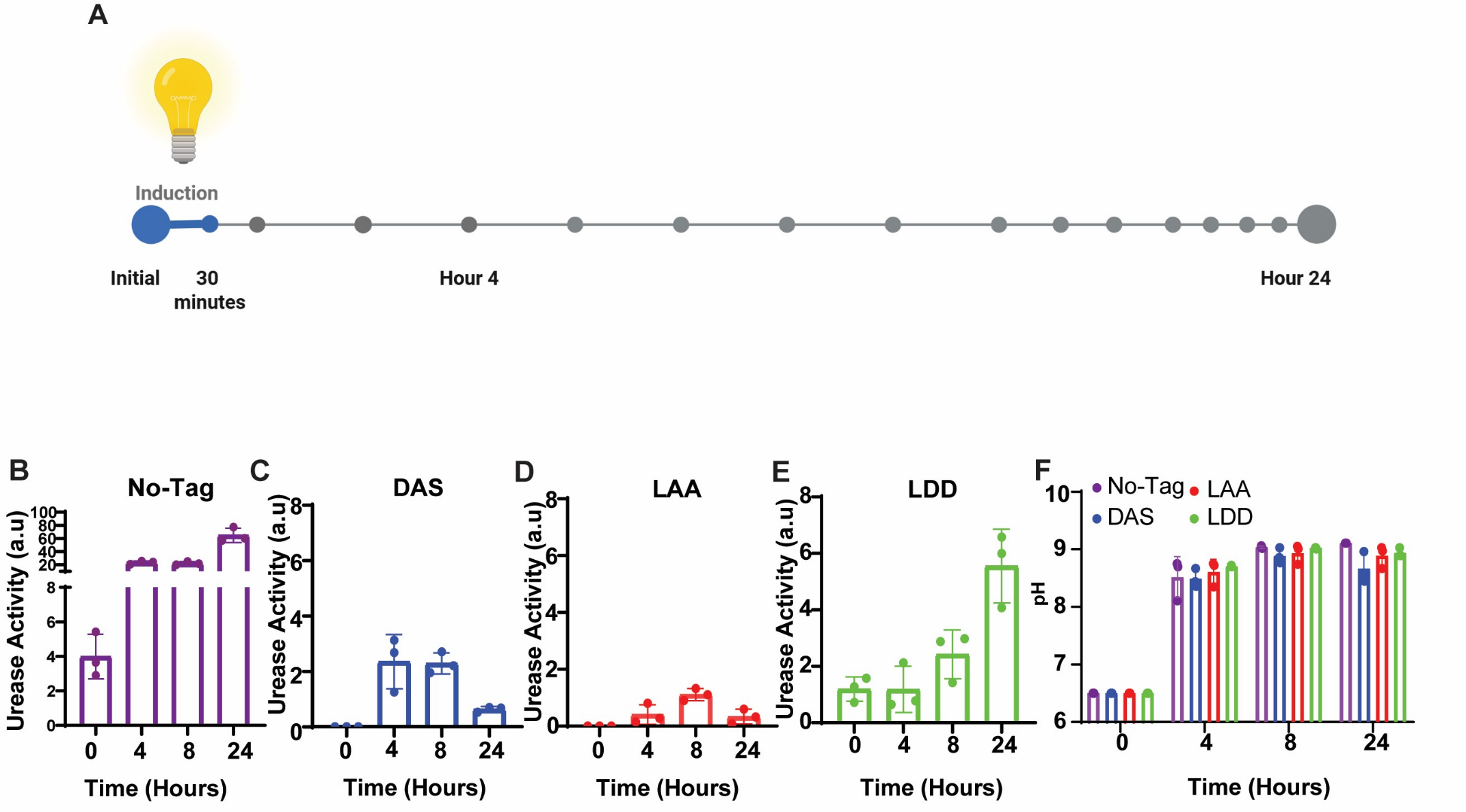


**Figure S4.** Effects of minimal irradiation on cultures expressing Urease-DAS fused to different degradation tags. **A**) Overview of experimental workflow involving 30 minutes of irradiation followed by 23.5 hours of culture in the dark. **B-E)** Urease activity measured independently of in situ culture pH at 0, 4, 8, 24 hours. **F)** In situ culture pH for cells urease fused to various degradation tags (No-tag, DAS, LAA, and LDD) at 0, 4, 8, 24 hours. Bars indicate mean ± standard deviation (n=3) replicates derived from the same starter culture.


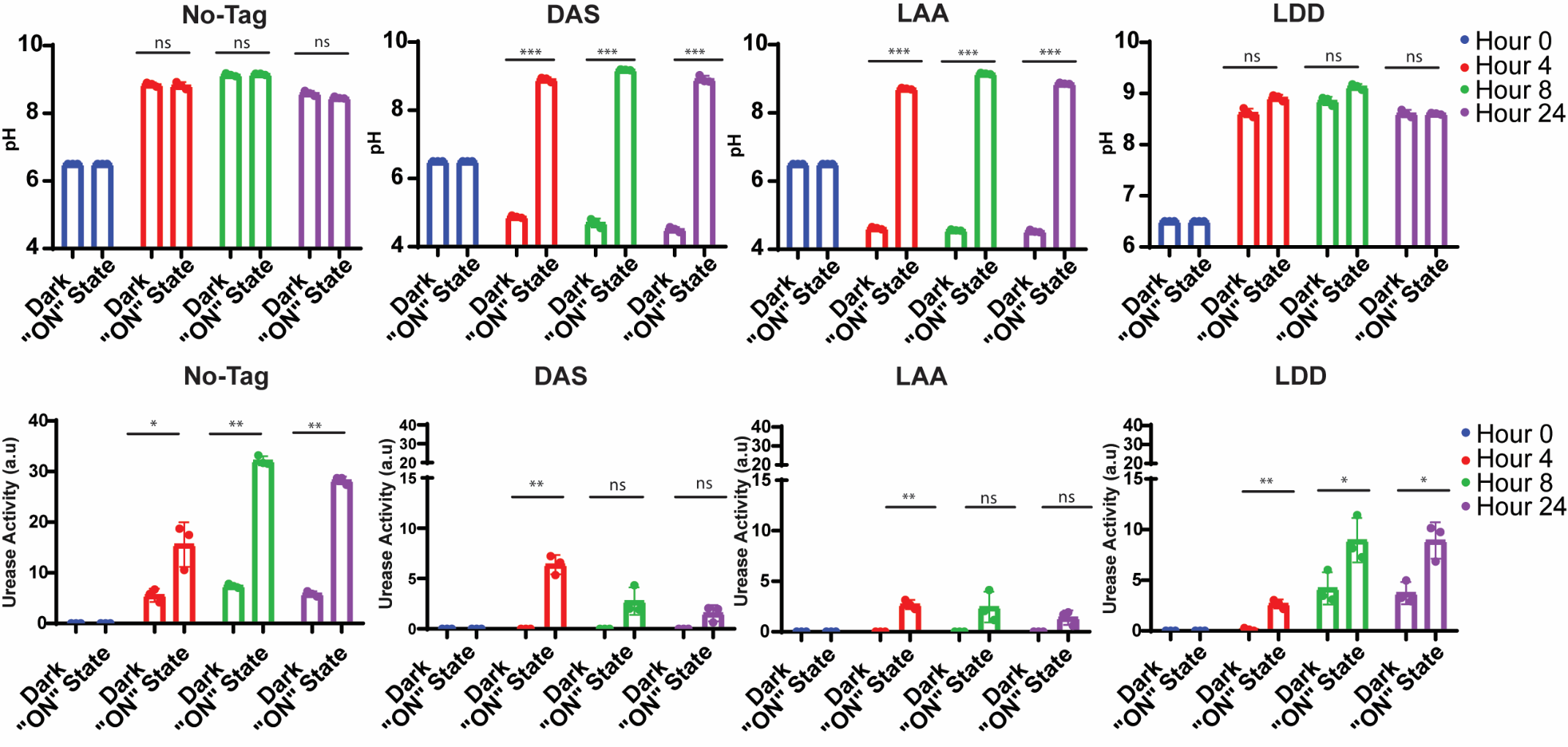


**Figure S5.** Statistical analysis for **Figure 1**. An unpaired two-tailed t-test was used to determine statistical significance between two groups (n = 3, ns, ∗p > 0.05, ∗∗p < 0.01, ∗∗∗p < 0.001). Bars indicate mean ± standard deviation (n=3) replicates derived from the same starter culture.


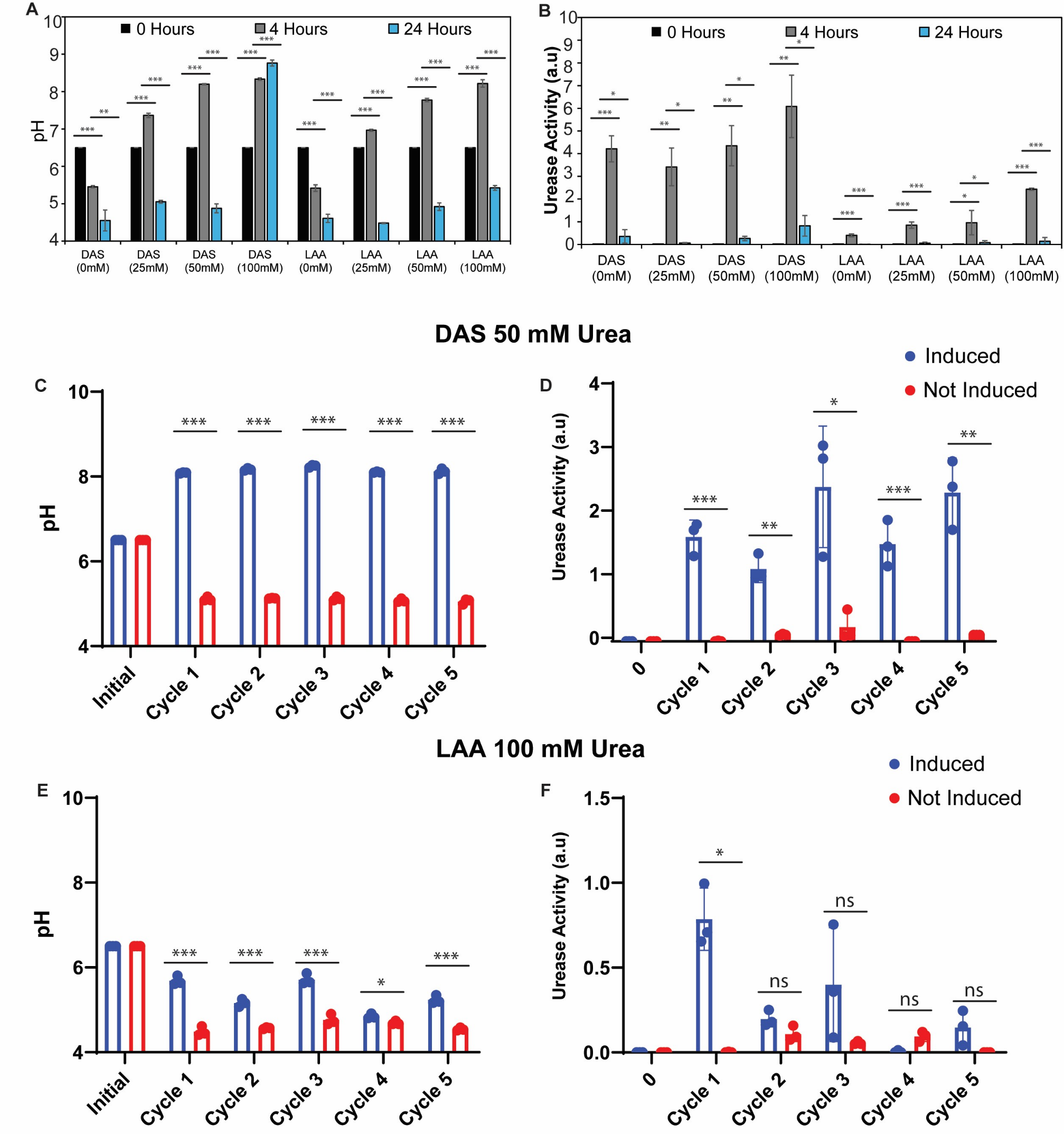


**Figure S6.** Statistical analysis for **Figure 3**. An unpaired two-tailed t-test was used to determine statistical significance between two groups (n = 3, ns, ∗p > 0.05, ∗∗p < 0.01, ∗∗∗p < 0.001). Bars indicate mean ± standard deviation (n=3) replicates derived from the same starter culture.


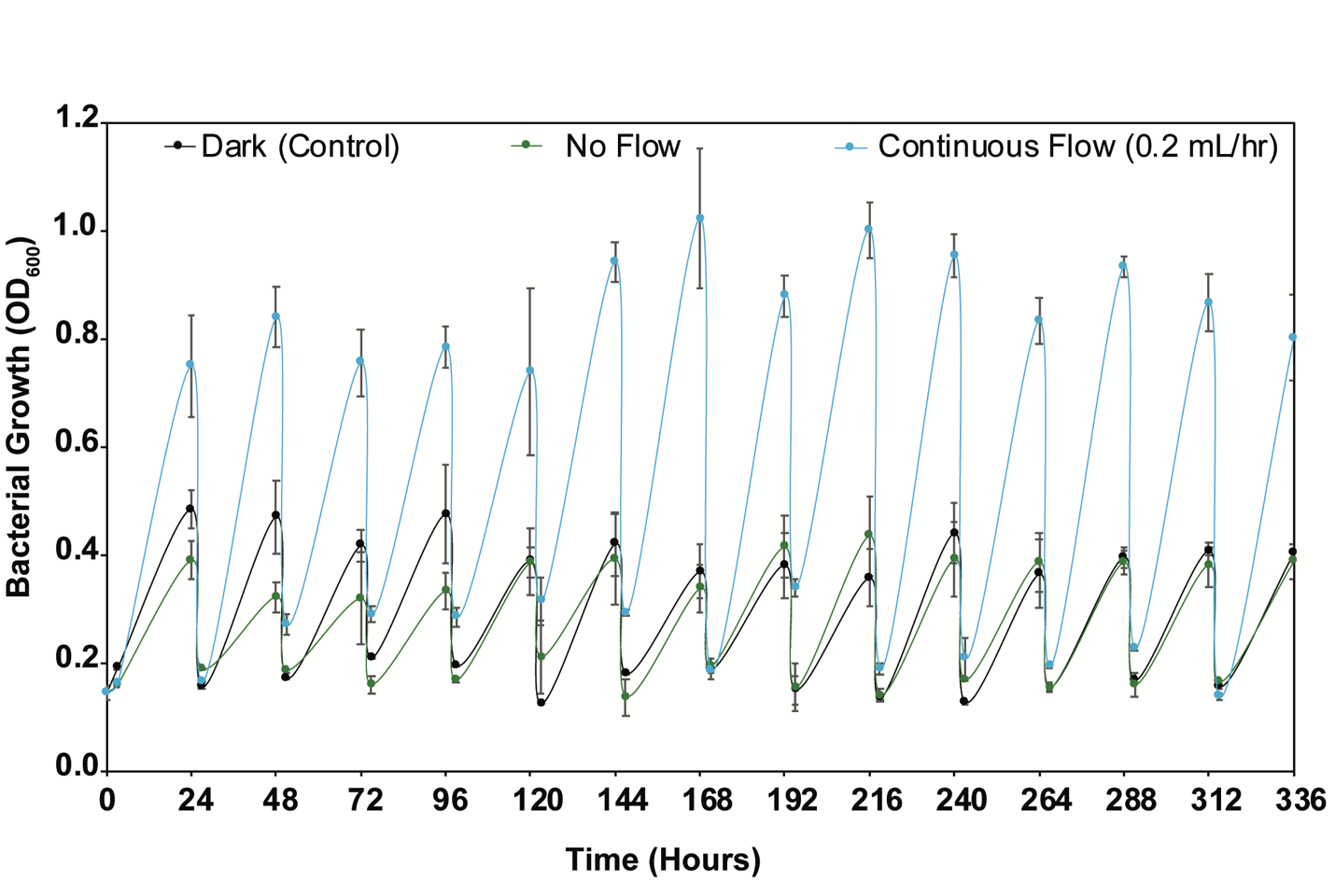


**Figure S7.** Growth of bacteria (OD_600_) expressing Urease-DAS over 14 days of basification and reacidification cycles. Data points show mean ± standard deviation (n=3) replicates derived from the same starter culture.


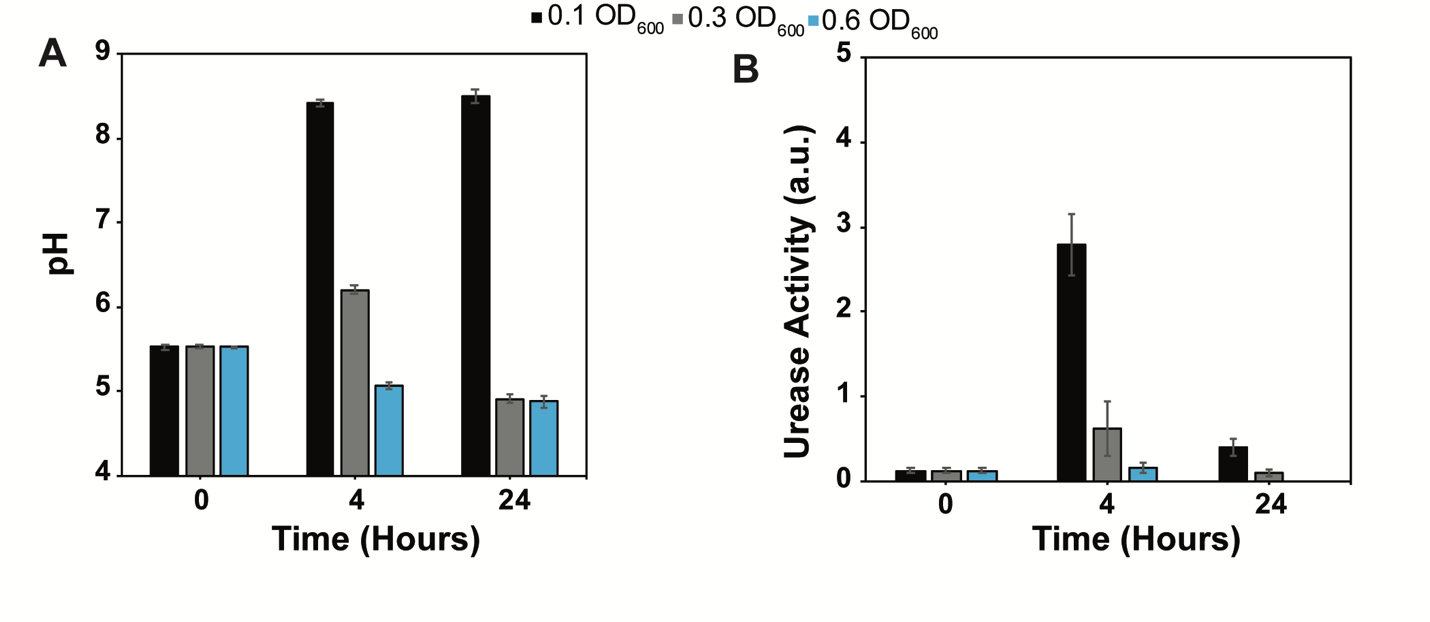


**Figure S8.** Effect of initial cell density on culture pH and intracellular urease activity in the continuous flow apparatus. In situ culture pH (**A)** and urease activity measured independently (**B)** after irradiation with 6 mW/cm^2^ blue light for 30 minutes. Measurements were taken 4- and 24-hours post-irradiation. Bars indicate mean ± standard deviation (n=3) replicates derived from the same starter culture.


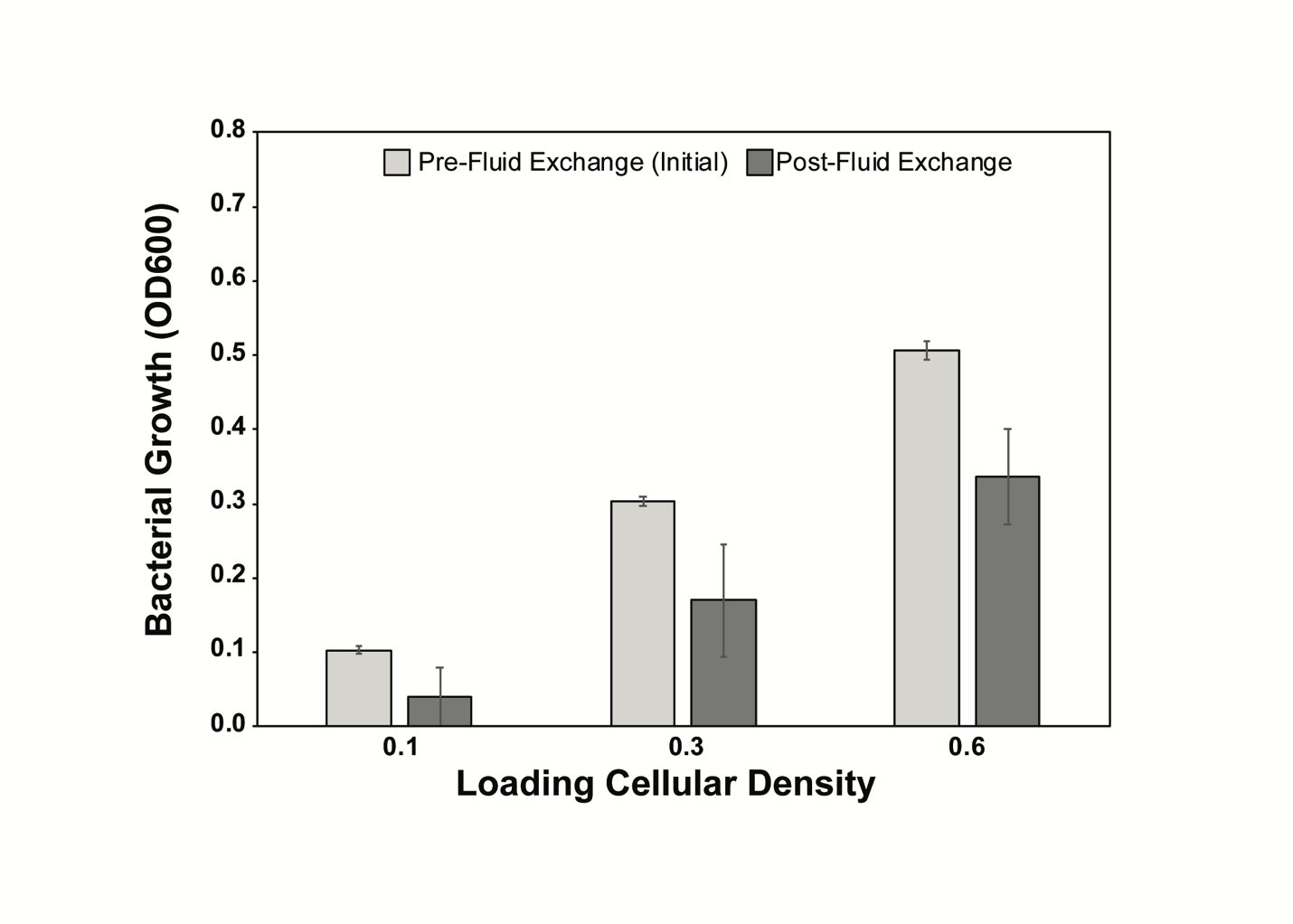


**Figure S9.** Effect of syringe pump-programmed flush (i.e., fluid exchange) on cell density inside well plate apparatus adapted for continuous flow. Normal operation of the continuous flow apparatus used a flow rate of 2 mL/hr, while the flush used a flow rate of 2 mL/min for 2 minutes. Cell density was measured by OD_600_ pre- and post-flush. Bars indicate mean ± standard deviation (n=3) replicates derived from the same starter culture.

**Table S4.** Bacterial strain name, description, and source of cell strain used in this study

| **Strain** | **Description** | **Source** |
| --- | --- | --- |
| BL21(DE3) | Commercially available protein expression *E. coli* strain | New England Biolabs, Inc. C2527H |
| DH10B | Commercially available protein expression *E. coli* strain | New England Biolabs, Inc. C3019H |
| Mach 1 | Commercially available *E. coli* strain designed for fast plasmid propagation | Thermo Fisher Scientific  #C862003 |

**Table S5.** Plasmid name, description, and source of plasmids used in this study

| **Plasmid** | **Description** | **Source** |
| --- | --- | --- |
| pAB202 | nMag and pMag under araB promoter | Addgene #101674 |
| pAB50 | mCherry under T7 promoter control | Addgene #101678 |
| pT7Opto Urease | Urease Complex under T7 promoter control | In this Study |
| pT7Opto DAS | Urease Complex with DAS degradation tag under T7 promoter control | In this Study |
| pT7Opto LAA | Urease Complex with LAA degradation tag under T7 promoter control | In this Study |
| pT7 Opto LDD | Urease Complex with LDD degradation tag under T7 promoter control | In this study |
| pPR220.08 | Gene containing PCB, cofactor for optogenetic regulation. UirS, sensor kinase gene | Addgene #78553 |
| pPR219.05 | Plamid containing UirR, response regulator and promoter machinery for GFP output | Addgene #78554 |

**Table S6.** Amino acid sequences of proteins in the urease enzyme complex, as encoded by the pOpto-T7 plasmid.

| **Gene** | **Sequence** |
| --- | --- |
| *ureA* | MHLNPAEKEKLQIFLASELALKRKARGLKLNYPEAVAIITSFIMEGARDGKTVAMLMEEGKHVLTRDDVMEGVPEMIDDIQAEATFPDGTKLVTVHNPIS* |
| *ureB* | MSNNNYIVPGEYRVAEGEIEINAGREKTTIRVSNTGDRPIQVGSHIHFVEVNKELLFDRAEGIGRRLNIPSGTAARFEPGEEMEVELTELGGNREVFGISDLTNGSVDNKELILQRAKELGYKGVE* |
| *ureC* | MKINRQQYAESYGPTVGDQVRLADTDLWIEVEKDYTTYGDEANFGGGKVLREGMGENGTYTRTENVLDLLLTNALILDYTGIYKADIGVKDGYIVGIGKGGNPDIMDGVTPNMIVGTATEVIAAEGKIVTAGGIDTHVHFINPDQVDVALANGITTLFGGGTGPAEGSKATTVTPGPWNIEKMLKSTEGLPINVGILGKGHGSSIAPIMEQIDAGAAGLKIHEDWGATPASIDRSLTVADEADVQVAIHSDTLNEAGFLEDTLRAINGRVIHSFHVEGAGGGHAPDIMAMAGHPNVLPSSTNPTRPFTVNTIDEHLDMLMVCHHLKQNIPEDVAFADSRIRPETIAAEDILHDLGIISMMSTDALAMGRAGEMVLRTWQTADKMKKQRGPLAEEKNGSDNFRAKRYVSKYTINPAIAQGIAHEVGSIEEGKFADLVLWEPKFFGVKADRVIKGGIIAYAQIGDPSASIPTPQPVMGRRMYGTVGDLIHDTNITFMSKSSIQQGVPAKLGLKRRIGTVKNCRNIGKKDMKWNDVTTDIDINPETYEVKVDGEVLTCEPVKELPMAQRYFLF* |
| *ureE* | MLITKIVGHIDDYESSDKKVDWLEVEWEDLNKRILRKETENGTDIAIKLENSGTLRYGDVLYESDDTLIAIRTKLEKVYVIKPQTMQEMGKMAFEIGNRHTMCIIEDDEILVRYDKTLEKLIDEVGVSYEQSERRFKEPFKYRGHQH* |
| *ureF* | METYIQESDISNEDDLKAFCDMYLRQNLASTDAIIAQEAYRLAKENDLQGLIRLENICHAIKLSPETRKGSMMMGRQFLQTVQPLNNSELFTIWCEKLKNKEIKSHYPVVYGIYTAMLGVDLRTSLETFLYSSITSLVQNGVRAIPLGQNSGVQTIFSLLPVIQETTSRVMTLDLEHLDNNSIGLEIASMKHEFLHSRLFIS* |
| *ureG* | MKTIHLGIGGPVGSGKTTLVKTLSEALKEEYSIAVITNDIYTREDANFLINENILEKDRIIGVETGGCPHTAIREDASMNFEAIEELKNRFDDLEIILLESGGDNLSATFSPELVDAFIYVIDVSEGGDIPRKGGPGVTRSDFLMVNKTELAPYVGVDLDTMKNDTIKARNGRPFTFANIKTKKGLDEIIAWIKSDLLLEGKTNESASESK* |
| *ureD* | MEFQYRGNKTVLSNCYQNPPLRASRPLYINPANRSEATVYLVETSGGIVEGDHNVFDIDIKEGADVCLIPQSATKIYPSYNGIWSSQDMDITIGPKASLSFKTEAVIPFEQARFNSKTVIQMTSDSTFLWGDILSPGRVARGEVFEYTDVRTNFQVWMDDECLIYDPLLISKDNMGLKKMGMLEDHLFIGSMWFVTPTIEEFDIRELNERLQESPHSKASASMLEGKAVNVRWLASDLVDLKKEMNRIWDEFANYIV* |
